# Sympathetic Dysautonomia in Heart Failure by ^123^I-MIBG: comparison between Chagasic, non-Chagasic and heart transplant patients

**DOI:** 10.5935/abc.20180124

**Published:** 2018-08

**Authors:** Viviane Santuari Parisotto Marino, Sandra Monetti Dumont, Luciene das Graças Mota, Daniela de Souza Braga, Stephanie Saliba de Freitas, Maria da Consolação Vieira Moreira

**Affiliations:** 1 Departamento de Anatomia e Imagem da Faculdade de Medicina da Universidade Federal de Minas Gerais, Belo Horizonte, MG - Brazil; 2 Hospital das Clínicas da Universidade Federal de Minas Gerais, Belo Horizonte, MG - Brazil; 3 Departamento de Clínica Médica da Faculdade de Medicina da Universidade Federal de Minas Gerais, Belo Horizonte, MG - Brazil

**Keywords:** Heart Failure, Primary Dysautonomies, Chagas Cardiomyopathy, Myocardial/radionuclide imaging, ^123^ I-metaiodobenzylguanidine ^123^I-MIBG)

## Abstract

**Background:**

Heart failure (HF) is a severe public health problem because of its high morbidity and mortality and elevated costs, thus requiring better understanding of its course. In its complex and multifactorial pathogenesis, sympathetic hyperactivity plays a relevant role. Considering that sympathetic dysfunction is already present in the initial phases of chronic Chagas cardiomyopathy (CCC) and frequently associated with a worse prognosis, we assumed it could be more severe in CCC than in cardiomyopathies of other etiologies (non-CCC).

**Objectives:**

To assess the cardiac sympathetic dysfunction ^123^I-MIBG) of HF, comparing individuals with CCC to those with non-CCC, using heart transplant (HT) patients as denervated heart parameters.

**Methods:**

We assessed 76 patients with functional class II-VI HF, being 25 CCC (17 men), 25 non-CCC (14 men) and 26 HT (20 men), by use of cardiac ^123^I-metaiodobenzylguanidine ^123^I-MIBG) scintigraphy, estimating the early and late heart-to-mediastinum ratio (HMR) of ^123^I-MIBG uptake and cardiac washout (WO%). The 5% significance level was adopted in the statistical analysis.

**Results:**

The early and late HMR values were 1.73 ± 0.24 and 1.58 ± 0.27, respectively, in CCC, and 1.62 ± 0.21 and 1.44 ± 0.16 in non-CCC (p = NS), being, however, higher in HT patients (p < 0.001). The WO% values were 41.65 ± 21.4 (CCC), 47.37 ± 14.19% (non-CCC) and 43.29 ± 23.02 (HT), p = 0.057. The late HMR values showed a positive weak correlation with left ventricular ejection fraction (LVEF) in CCC and non-CCC (r = 0.42 and p = 0.045; and r = 0.49 and p = 0.015, respectively).

**Conclusion:**

Sympathetic hyperactivity ^123^I-MIBG) was evidenced in patients with class II-IV HF, LVEF < 45%, independently of the HF etiology, as compared to HT patients.

## Introduction

Heart failure (HF) currently represents a public health problem because of its epidemic proportions (worldwide prevalence greater than 23 million individuals), its high morbidity and mortality, and, consequently, high health expenditures.^1^

A large variety of cardiac conditions can result in HF, whose prevalence differs when comparing developed and developing countries.^2^ In Brazil, the ischemic etiology accounts for 34.1% of the HF cases, being followed by the Chagasic (21.4%) and hypertensive (13.2%) etiologies, the Chagasic being associated with a worse prognosis.^3^^-^^6^

Previous studies have suggested that the pathogenesis of HF is complex and multifactorial,^7^ sympathetic dysautonomia playing a relevant role in the process.^8^^-^^11^ In the initial phase of HF, the sympathetic nervous system activation would modulate the pump function; however, over time, its action would become deleterious, leading to myocardial remodeling and restructuring, with progressive decline of the cardiac function.^10^^,^^12^^,^^13^ In such patients, sympathetic dysfunction would be characterized by a significant reduction in the presynaptic uptake of norepinephrine, with consequent elevation in its serum levels and reduction in the postsynaptic density of b-adrenoreceptors.^10^^,^^13^^,^^14^

In chronic Chagas cardiomyopathy (CCC), early impairment of the parasympathetic nervous system has been well established,^15^^,^^16^ and sympathetic dysfunction, although not totally established, is believed to be present in the initial phases of the disease,^11^^,^^17^^-^^19^ when the cardiac pump function is preserved and potentially associated with malignant arrhythmias and sudden death.^20^^-^^22^

Because cardiac autonomous dysfunction is present early in CCC and the incidence of malignant arrhythmias and sudden death is elevated in those patients,^15^^,^^16^^,^^20^^-^^22^ this study was designed aimed at assessing the presence and the magnitude of cardiac sympathetic dysfunction in Chagasic patients with HF. Chagasic *versus* non-Chagasic patients were compared, and heart transplant (HT) patients were considered as the denervated heart pattern (known to be abnormal).^23^ Cardiac ^123^I-metaiodobenzylguanidine (^123^I-MIBG) scintigraphy was used to assess the patients, because it properly evaluates cardiac sympathetic dysfunction,^12^^,^^13^^,^^24^^,^^25^ providing relevant parameters to understand the progression of HF.^12^^,^^13^^,^^24^

## Methods

This is a cross-sectional study of 76 patients selected from the Heart Failure and Heart Transplant outpatient clinic of our institution from March 2014 to February 2016.

The eligibility criteria for individuals with HF were: age over 18 years; left ventricular ejection fraction (LVEF) ≤ 45%; confirmed non-Chagasic or Chagasic etiology (positivity for Chagas disease confirmed by use of two different serological techniques) associated with left ventricular systolic dysfunction;^26^ and accepting to participate in the study. In addition, for individuals with HF submitted to HT (comparison group or denervated heart model),^23^ time from HT shorter than 12 months was required. Patients with diabetes mellitus, chronic kidney disease, chronic obstructive pulmonary disease, Parkinson disease, non-sinus heart rhythm or implantable pacemaker were excluded.

The patients were studied prospectively, divided into three groups: CCC group - 25 patients with CCC (mean age, 53.3 ± 9.2 years; 17 males); non-CCC group - 25 patients with heart disease etiologies other than CCC (56% idiopathic, 36% ischemic, and 8% post-partum cardiomyopathy; mean age, 43.3 ± 12 years; 14 males); and HT group - 26 patients previously submitted to HT within less than 12 months (mean, 6.5 ± 3.8 months), with mean age of 47.3 ± 13.1 years, being 20 of the male sex. All patients provided written informed consent, which had been approved by the Ethics Committee of the institution, according to the Declaration of Helsinki. All patients underwent clinical control during the study period.

**Figure 1:**
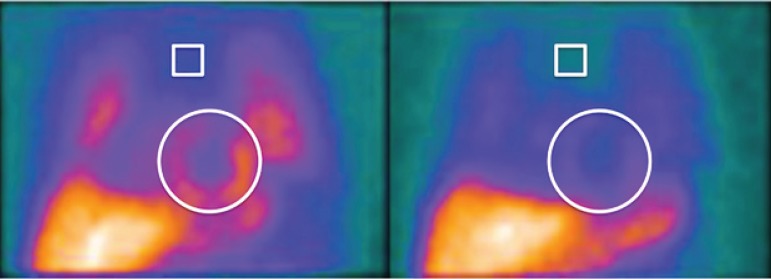
Early (15-minute) and late (180-minute) anterior planar imaging of the chest by 123I-MIBG scintigraphy, with regions of interest (ROI) positioned on the superior mediastinum between the pulmonary fields and heart.

Clinical, electrocardiographic (ECG at rest) and echocardiographic data were collected by the same researcher. Echocardiography was performed using the Phillips iE33® ultrasound device (Phillips Medical, Andover, MA, USA), LVEF being estimated by using Simpson's formula.^27^ Planar scintigraphy of the myocardial innervation was performed by use of slow intravenous administration of 111 MBq/3 mCi ^123^I-MIBG (IPEN/CNEN), with anterior image acquisition of the chest after 15 minutes and 180 minutes on a Hawkeye® gamma-camera (GE healthcare, Milwaukie, USA), 10 min/frame, ^123^I photopeak of 159 KeV, window of 20%, and low-energy high-resolution collimator (LEHR). The heart region of interest (ROI) was drawn encompassing the entire left ventricle, while that of the superior mediastinum encompassed a square ROI of 12x12 pixels. Early and late cardiac uptakes were estimated by use of the ratio between the radioactive counts of the heart and mediastinal ROIs on early and late imaging (early HMR and late HMR, respectively). The cardiac washout rate (WO%) of ^123^I-MIBG was calculated using the formula: (early heart uptake early mediastinal uptake) (late heart uptake late mediastinal uptake) / (early heart uptake early mediastinal uptake) x 100, without considering radioactive decay, and expressed as percentages^28^ (Figure 1). Two nuclear physicians analyzed separately the images, with 98% of interobserver agreement, and defined the following as abnormal: WO% > 27% and late HMR ≤ 1.8.^29^

The effective radiation dose for the patient, resulting from the administration of 111 MBq/3mCi of ^123^I-MIBG was estimated as approximately 4.8 mSv, comparable to one of the phases of myocardial perfusion studies with ^99m^Tc-isonitrile.^30^

### Statistical analysis

For this analysis, a sample of 76 patients was calculated to detect a 12% variation in the early or late ^123^I-MIBG uptake (HMR), with 5% alpha error and 80% power (CI = 95%) for three groups of patients.

To characterize the sample, descriptive analysis of the following variables was initially performed: sex, age, heart rate (HR), LVEF, early HMR, late HMR and WO% of ^123^I-MIBG expressed according to the distribution of frequency or measures of central tendency and variability. This analysis was stratified per group (CCC, non-CCC and HT). When comparing the three groups, for the categorical variables (sex, HR, NYHA functional class, use of beta-blockers), Pearson chi-square test was performed; for the continuous variables (age, LVEF, WO%), one-factor analysis of variance (ANOVA) was used; and for multiple comparisons, the least significant difference (LSD) test was used. In addition, ANOVA was used to assess the variables (early and late HMR), based, however, on repeated-measures analysis and LSD test for multiple comparisons.

It is worth noting that the assumptions to use ANOVA were verified and accepted, that is, normally distributed residuals (Kolmogorov-Smirnov test) and constant variances (Levene's test).

To analyze the correlation between the measures of early HMR, late HMR or WO% and LVEF, Pearson correlation test and its respective p value were used. In all analyses, a 5% significance level was considered, and the statistical software SPSS, version 17.0 (SPSS Inc., Illinois, USA), was used.^31^^,^^32^

## Results

Table 1 shows the demographic, clinical and echocardiographic data of the patients studied. Those with HF on angiotensin-converting-enzyme inhibitors (ACEI) and beta-blockers maintained their medications. In approximately 70% of the HT patients, HR was maintained over 80 bpm (mean of 90.7 bpm), while in individuals with CCC and non-CCC, the mean HR values were 72.7 bpm and 75.6 bpm, respectively (p = 0.03). No patient was on tricyclic antidepressants.

**Table 1:** Demographic, clinical and echocardiographic characteristics of the patients

	CCC	non-CCC	HT	p
Male sex^†^	68.0	56.0	77.0	0.281**^a^**
Age (years)*	53.3 ± 9.2	43.3 ± 12	47.3 ± 13.1	0.016**^c^**
HR > 80 bpm^†^	30.8	33.3	69.2	0.072**^a^**
NYHA II-IV^†^	62.5	92.0	0.0	< 0.001**^a^**
LVEF % (Echo)*	30.6 ± 7.8	25.9 ± 8.0	66.6 ± 8.3	< 0.001**^c^** CCC = non-CCC < HT
ACEI^†^	91.3	88	77.3	0.394**^b^**
Beta-blockers^†^	91.3	100	18.2	< 0.001**^a^** CCC = non-CCC > HT

CCC: chronic Chagas cardiomyopathy; non-CCC: cardiomyopathy other than Chagas disease; HT: heart transplant; HR: heart rate (bpm); NYHA: New York Heart Association (heart failure functional classification); ACEI: angiotensin-converting-enzyme inhibitors; Echo: echocardiography;

(*) expressed as mean and standard deviation;

(†) expressed as percentage. Note: The probability of statistical significance for the comparison of the groups refers to

(a) chi-square test,

(b) Fisher exact test, and

(c) analysis of variance.

**Figure 2:**
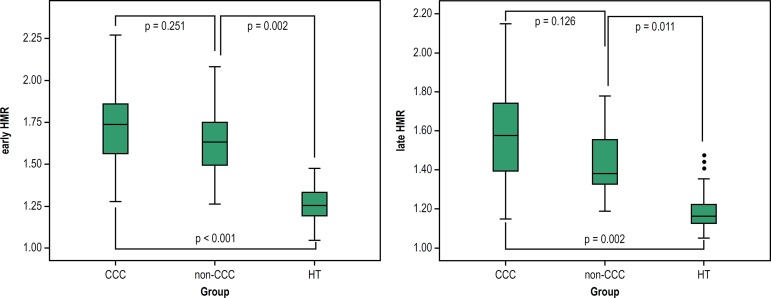
Early and late HMR of 123I-MIBG in CCC or non-CCC or HT patients. Early HMR - ratio of the heart/mediastinum radioactive counts estimated on 15-minute images (early uptake); late HMR: ratio of the heart/mediastinum radioactive counts estimated on 180-minute images (late uptake). Groups: CCC: chronic Chagas cardiomyopathy; non-CCC: cardiomyopathy etiology other than Chagas disease; HT: heart transplant.

The early and late HMR values were greater than those reported for HT patients (p < 0.001) (Table 2), but did not differ in CCC or non-CCC patients with HF, even when adjusted for age and age group (early HMR: p = 0.251; and late HMR: p = 0.011). The early HMR values of CCC patients were 8.6% higher than those found in non-CCC patients, and 39.7% higher than those found in HT patients. The late HMR values of CCC patients were 9.7% higher than those of non-CCC patients, and 31.7% higher than those of HT patients (Figure 2).

**Table 2:** Scintigraphic parameters of myocardial dysfunction (^123^I-MIBG) in CCC, non-CCC and HT patients

^123^I-MIBG	CCC	non-CCC	HT	p
Early HMR*	1.73 ± 0.24	1.62 ± 0.21	1.26 ± 0.10	< 0.001**^a^**
Late HMR*	1.58 ± 0.27	1.44 ± 0.16	1.20 ± 0.12	< 0.001**^a^**
WO%	41.60 ± 21.41	47.37 ± 14.19	43.29 ± 23.02	0.057**^b^**

CCC: chronic Chagas cardiomyopathy; non-CCC: cardiomyopathy other than Chagas disease; HT: heart transplant; early HMR: ratio of the heart/mediastinum radioactive counts estimated on 15-minute images (early uptake); late HMR: ratio of the heart/mediastinum radioactive counts estimated on 180-minute images (late uptake); WO%: cardiac washout of ^123^I-MIBG, expressed as percentage;

(*) values expressed as mean and standard deviation. Note: The probability of statistical significance refers to analysis of variance based on

(a) repeated measures and

(b) analysis of variance.

**Figure 3:**
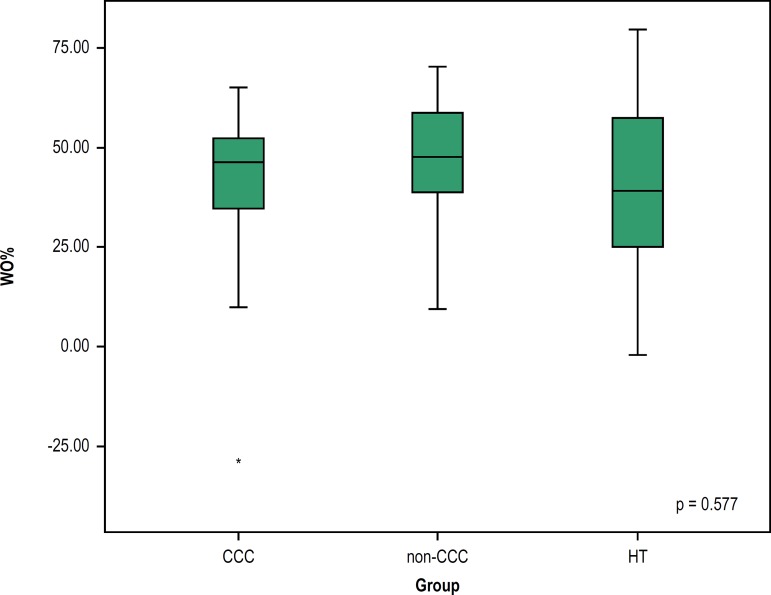
Cardiac washout (WO%) of ^123^I-MIBG in CCC or non-CCC or HT patients. Groups: CCC: chronic Chagas cardiomyopathy; non-CCC: cardiomyopathy etiology other than Chagas disease; HT: heart transplant.

The WO% values showed no statistically significant difference between individuals with HF, and between individuals with HF and those submitted to HT (p = 0.577) (Figure 3).

**Figure 4:**
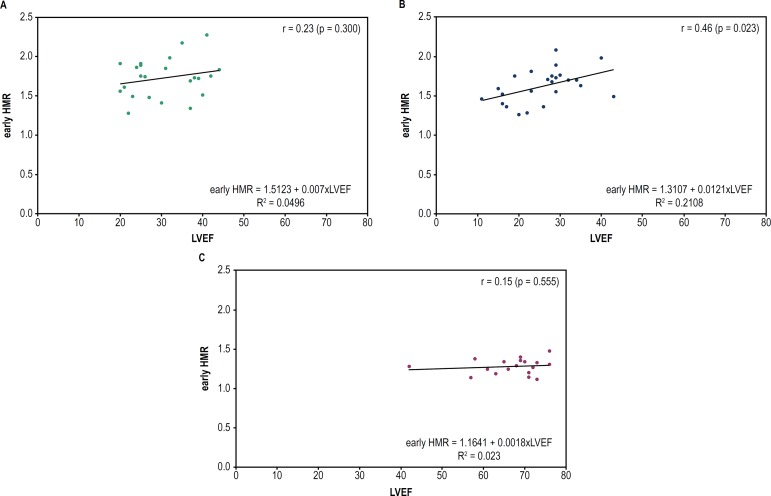
Dispersion graph to assess the correlation between LVEF and early HMR in CCC or non-CCC or HT patients. Early HMR: ratio of the heart/mediastinum radioactive counts estimated on 15-minute images (early uptake); LVEF: left ventricular ejection fraction. (A) CCC: chronic Chagas cardiomyopathy; (B) non-CCC: cardiomyopathy etiology other than Chagas disease; (C) HT: heart transplant.

**Figure 5:**
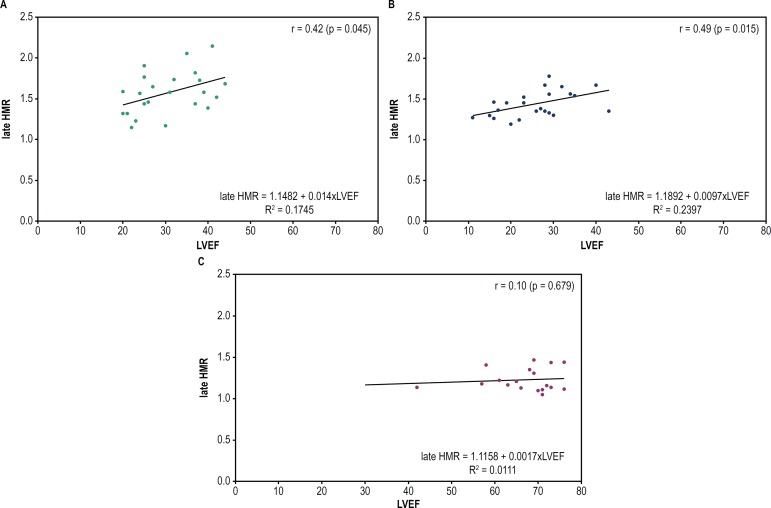
Dispersion graph to assess the correlation between LVEF and late HMR in CCC or non-CCC or HT patients. Late HMR: ratio of the heart/mediastinum radioactive counts estimated on 180-minute images (late uptake); LVEF: left ventricular ejection fraction. (A) CCC: chronic Chagas cardiomyopathy; (B) non-CCC: cardiomyopathy etiology other than Chagas disease; (C) HT: heart transplant.

**Figure 6:**
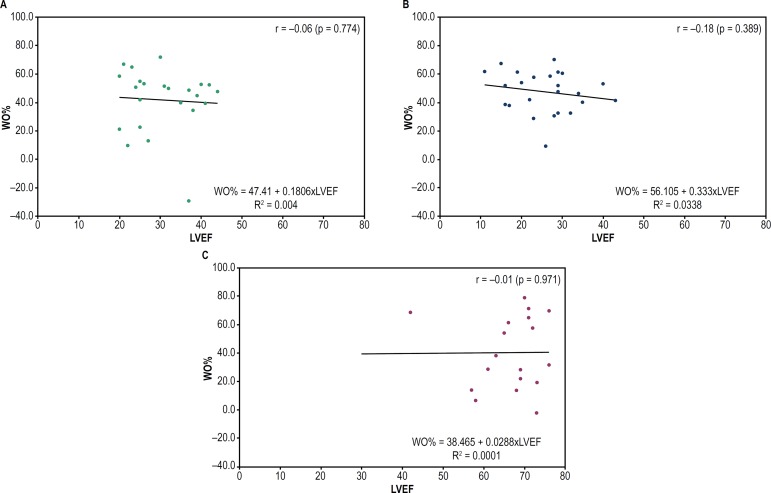
Dispersion graph to assess the correlation between LVEF and WO% of 123I-MIBG in CCC or non-CCC or HT patients. CCC: chronic Chagas cardiomyopathy; LVEF: left ventricular ejection fraction. (A) CCC: chronic Chagas cardiomyopathy; (B) non-CCC: cardiomyopathy etiology other than Chagas disease; (C) HT: heart transplant.

A weak positive correlation was observed between late HMR values and LVEF in CCC patients (r = 0.42; p = 0.045). Regarding the non-CCC patients, a positive correlation was observed both between LVEF and early HMR (r = 0.46; p = 0.023) and between LVEF and late HMR (r = 0.49; p = 0.015). However, none of the groups showed a correlation between LVEF and WO% (Figures 4, 5 and 6).

## Discussion

This study investigated the presence and magnitude of cardiac dysautonomia in patients with HF and LVEF ≤ 45% by use of ^123^I-MIBG scintigraphy. Patients were divided into three groups, CCC, non-CCC and HT, the latter, by representing the denervated heart model, served as the abnormality pattern.^23^

There was scintigraphic evidence of sympathetic hyperactivity, based on the findings of low ^123^I-MIBG uptake (early and late HMR) by the presynaptic endings in the three groups studied, which is aligned with the literature.^13^^,^^23^^,^^24^^,^^28^ The low ^123^I-MIBG uptake indicates dysfunction of the receptors and integrity loss of the presynaptic sympathetic fibers, reinforcing the theory of sympathetic hyperactivity in the pathogenesis of HF.^8^^-^^10^^,^^12^^,^^24^

Scintigraphy is the only noninvasive and safe method, sufficiently sensitive to assess the autonomic sympathetic nervous system,^12^^,^^24^ that can provide parameters known for their accuracy and reproducibility to estimate the efficacy of clinical treatment^13^ and the prognosis of patients with HF.^13^^,^^24^^,^^25^^,^^33^ However, the lack of standardization in the scintigraphic imaging acquisition and processing hinders the incorporation of the method into clinical practice, because there is no well-defined reference value.^28^^,^^29^ In a meta-analysis of seven studies with 96 healthy individuals, Patel and Iskandrian have reported HMR of 2.13 ± 0.3 and WO% of 20 ± 10% (ranging from 10 ± 6% to 37 ± 5%) for healthy individuals.^29^

The literature on HF has reported reduced late HMR values (1.80),^24^ with a correlation between a reduction in uptake and worse prognosis, expressed as a higher number of cardiac events and higher mortality.^13^^,^^24^^,^^25^ The late HMR values found for our patients with HF (Table 2) were lower than those adopted by different authors using cutoff point values < 1.75 (sensitivity of 84% and specificity of 60%),^13^ < 1.68,^25^ or even, more restrictive, < 1.60.^24^

The HMR values found for HT patients (1.20 ± 0.12) were lower than those found for individuals with HF, with statistical significance (p<0.001), which is aligned with that reported in the literature for patients within the first post-HT year, specially individuals with idiopathic heart disease.^23^^,^^29^

It is worth noting that the patients of this study were on regular use of beta-blockers and ACEI, which do not interfere directly in the uptake of noradrenaline. However, by improving the cardiac performance, and, thus, the sympathetic tone, they increment the ^123^I-MIBG uptake.^34^ Thus, supposedly, the HMR values of our patients are overestimated, reinforcing their sympathetic dysfunction degree.

Considering that the late HMR values of individuals with CCC (1.58 ± 0.27) are overestimated, and that Gadioli et al.^21^ have reported a significant correlation between late HMR values of 1.68 ± 0.19 and severe ventricular arrhythmias, we assumed that the sympathetic dysfunction was more severe in the CCC group because of its arrhythmic findings as compared to the non-CCC group.^20^^,^^22^ However, in our study, those values did not differ significantly from those of the non-CCC group (1.44 ± 0.16), even when statistically adjusted to age and LVEF (p = 0.111).

This fact might be explained by the advanced HF stage of our patients, when sustained autonomic sympathetic dysfunction represents a deleterious mechanism in the pathogenesis of HF itself,^8^^-^^10^^,^^13^^,^^24^^,^^25^^,^^33^ independently of etiology, which is aligned with the report by other authors.^13^

On the other hand, the importance of sympathetic dysfunction in CCC has been questioned by different authors because of: variation in the intensity of denervation; absence of correlation between parasympathetic denervation and myocardial dysfunction extent;^19^ presence of autonomic dysfunction in the early stages of Chagas disease;^4^^,^^15^^,^^19^ correlation between persistence of the myocardial inflammatory process and those patients' morbidity and mortality,^35^ despite the serum levels of catecholamines.

The estimated WO% values were elevated and abnormal in the three groups studied, being compatible with sympathetic dysfunction in patients with HF and not different from those of HT (p = 0.577). The sympathetic tone, translated by WO%,^13^^,^^28^ might be altered earlier and more markedly than late HMR, and, thus, could be a more sensitive parameter for prognostic assessment, as described.^33^

Finally, a positive, although weak, but statistically significant correlation between HMR and LVEF was observed in individuals with HF. The LVEF is routinely used for the prognostic assessment of HF.^22^^,^^36^ Thus, that weak correlation can indicate that this scintigraphic parameter is more accurate and earlier altered, as reported by Ogita et al., suggesting it is a better predictor of prognosis than LVEF.^33^ In addition, it is worth noting that the correlation of early and late HMR with LVEF in CCC patients identified in this study has not been reported in the literature.

### Study limitations

This is a cross-sectional nonrandomized study of patients with advanced HF (NYHA functional class II-IV), thus its findings cannot be generalized to all individuals with CCC. There are no strictly established reference values to quantify the scintigraphic parameters because different methodologies have been used (decay factor, correction of septal penetration of iodine).^28^^,^^29^ The maintenance of the medication by the patients (ACEI and beta-blockers) might have led to overestimation of the HMR values and influenced our results, although the number of patients on medications did not differ between the CCC and non-CCC groups, and most studies in the literature have been performed considering the severity of HF.^24^^,^^25^^,^^33^

## Conclusion

This study evidenced the presence of cardiac sympathetic autonomic dysfunction on myocardial ^123^I-MIBG scintigraphy, regardless of the HF etiology, and its magnitude was equal in individuals with CCC and non-CCC as compared to HT patients.
